# Incubation temperature impacts nestling growth and survival in an open‐cup nesting passerine

**DOI:** 10.1002/ece3.3911

**Published:** 2018-02-19

**Authors:** Emilie A. Ospina, Loren Merrill, Thomas J. Benson

**Affiliations:** ^1^ Illinois Natural History Survey Prairie Research Institute University of Illinois at Urbana‐Champaign Champaign IL USA; ^2^ Department of Natural Resources and Environmental Sciences University of Illinois at Urbana‐Champaign Urbana IL USA

**Keywords:** carry‐over effects, early‐life experiences, embryonic development, incubation, temperature manipulation

## Abstract

For oviparous species such as birds, conditions experienced while in the egg can have long‐lasting effects on the individual. The impact of subtle changes in incubation temperature on nestling development, however, remains poorly understood, especially for open‐cup nesting species with altricial young. To investigate how incubation temperature affects nestling development and survival in such species, we artificially incubated American robin (*Turdus migratorius*) eggs at 36.1°C (“Low” treatment) and 37.8°C (“High” treatment). Chicks were fostered to same‐age nests upon hatching, and we measured mass, tarsus, and wing length of experimental nestlings and one randomly selected, naturally incubated (“Natural”), foster nest‐mate on days 7 and 10 posthatch. We found significant effects of incubation temperature on incubation duration, growth, and survival, in which experimentally incubated nestlings had shorter incubation periods (10.22, 11.50, and 11.95 days for High, Low, and Natural eggs, respectively), and nestlings from the Low treatment were smaller and had reduced survival compared to High and Natural nestlings. These results highlight the importance of incubation conditions during embryonic development for incubation duration, somatic development, and survival. Moreover, these findings indicate that differences in incubation temperature within the natural range of variation can have important carryover effects on growth and survival in species with altricial young.

## 1 INTRODUCTION

Environmental conditions during development can impact both short‐ and long‐term phenotypic expressions (Weaver, 2009). Indeed, the thermal regime an organism is exposed to during development has been shown to influence short‐ and long‐term phenotypic expression, including survival, hatchling size, posthatch growth, locomotor performance, morphology, and sex differentiation (Andrews, Mathies, & Warner, 2000; Bull, 1980; Rana, 1990; Shine, Elphick, & Harlow, 1997; Van Damme, Bauwens, Braña, & Verheyen, 1992). For oviparous species, embryonic development occurs outside of the mother once incubation is initiated. Embryos are particularly sensitive to environmental factors such as temperature, and proper development occurs within a narrow range of temperatures (Webb, 1987). Consequently, conditions experienced while in the egg can have important and potentially long‐lasting effects on individuals. Carryover effects of incubation temperature have been reported in desert tortoises (*Gophers agassizii*) in which incubation temperatures resulted in poor‐condition hatchlings that died within 45 days of hatching (Spotila et al., 1994). Similarly, Burger (1989, 1990) demonstrated carryover effects of incubation temperature on whole‐animal functions such as striking and escape behaviors as well as maneuverability and locomotion in young pine snakes (*Pituophis melanoleucus*), black racers (*Coluber constrictor*), and kingsnakes (*Lampropeltis getulus*), in which both high and low incubation temperatures negatively impacted young.

Unlike most other oviparous taxa in which organisms select an appropriate thermal environment for the eggs and rely on site selection for incubation, over 99% of bird species take an active role in incubation (Deeming, 2002a, 2002b). In all species of birds, incubation temperatures are in some fashion mediated by actions of the adults, including nest location, nest structure, and incubation behavior (Deeming, 2002a, 2002b). These behaviors can be broadly classified as “parental effects,” defined as the influence parents have on the expression of the offspring's phenotype that is unrelated to the offspring's genotype (Mousseau & Fox, 1998; Uller, 2008). Parental effects can be broken down into paternal and maternal effects, and in general, females have greater capacity to exert parental effects, especially during the prehatch phase of development.

For many bird species, incubation is performed solely by the female and thus represents a maternal effect. The incubation period plays a crucial role in reproduction, with most species exhibiting contact incubation to maintain a suitable environment for embryonic growth and development (Deeming, 2002a, 2002b). During this period, adult birds must balance current reproductive expenditures (e.g., demands of the developing embryo) against future reproductive potential (e.g., costs of self‐maintenance) (Reid, Monaghan, & Nager, 2002; Stearns, 1992) as well as energetic expenditures resulting from a trade‐off between maintaining proper incubation conditions for the developing embryo and foraging needs of the adult(s) (Skutch, 1962). Despite the variety of climates in which eggs are laid, avian embryonic development is generally restricted to a thermal range between 30.0 and 40.0°C (Webb, 1987), but for many species, the optimal range is substantially smaller (38.0–39.0°C; Carey, 1980). Prolonged exposure to temperatures outside of this optimal range can be deleterious for developing embryos (Lundy, 1969) of many species. Nest attentiveness is likely the primary mode by which birds mediate incubation temperatures, and decreases in nest attentiveness are known to impact nestling development and survival in a number of ways, including: (1) extending the development period, thereby prolonging the time a nest is susceptible to predation (Martin, 2002), (2) reducing hatching success and survival of young (DuRant, Hepp, Moore, Hopkins, & Hopkins, 2010), and (3) retarding morphological development of nestlings (Nord & Nilsson, 2011; Webb, 1987). There has been substantial work on the effects of incubation temperature in the poultry industry related to various aspects of pre‐ and posthatch development and physiology (e.g., Hulet, Gladys, Hill, Meijerhod, & El‐Shiekh, 2007; Leksrisompong, Romero‐Sanchez, Plumstead, Bannan, & Brake, 2007; Michels, Geers, & Muambi, 1974; Nangsuay et al., 2016); however, research on wild birds is more limited. Much of the work on incubation temperature in wild birds has focused on cavity‐nesting species, and/or species with precocial offspring (DuRant et al., 2010; Hepp, Kennamer, & Johnson, 2006; Nord & Nilsson, 2011), primarily because species with these life‐history traits are amenable to temperature manipulation studies. However, the majority of bird species are open‐cup nesting species with altricial young, and to date, little work has been done examining how changes in incubation temperature impact development and survival of open‐cup nesting species with altricial young (but see: Ton & Martin, 2017). While studies on cavity‐nesting species have provided critical insight into the importance of incubation temperature for proper growth and development (Ardia, Pérez, & Clotfelter, 2010; Wada et al., 2015), there are a number of important differences between these species and those that build open‐cup nests and have altricial young. With respect to nest type, species with altricial young that use nest cavities take frequent foraging trips and have relatively short on‐ and off‐bouts during the incubation period compared to open‐cup nesting species (Conway & Martin, 2000). The combination of short off‐bout periods and the more thermally insulated environment of a cavity are thought to result in smaller temperature fluctuations for the developing eggs. Open‐cup nesting birds are exposed to higher predation rates (Lack, 1954; Nice, 1957; Ricklefs, 1969) and greater variability in temperature (relative to enclosed nests; Martin et al., 2017) throughout the nesting period (Heenan, 2013). As such, open‐cup nesting species producing altricial young may be more sensitive to changes in incubation temperatures as compared to precocial and/or cavity‐nesting species, which may profoundly impact growth, development, and survival.

**Figure 1:**
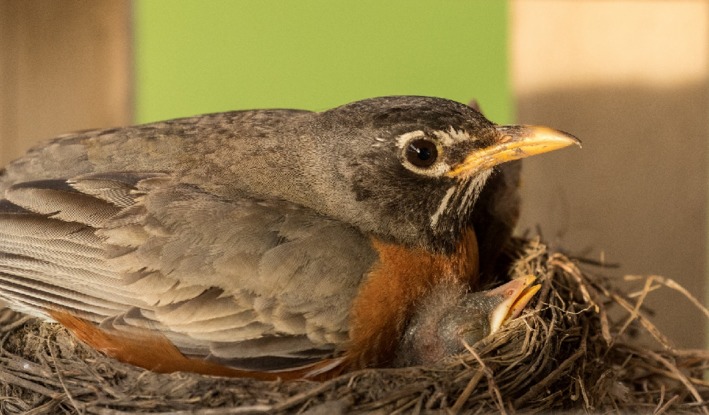
Adult American robin (*Turdus migratorius*) brooding nestlings, Champaign, IL, USA. Photograph by Loren Merrill

To better understand how variation in incubation conditions impacts the length of the incubation period, hatching success, nestling development, and survival of an open‐cup nesting species with altricial young, we artificially incubated eggs of the American robin (*Turdus migratorius*: hereafter referred as robin(s); Figure 1) at 36.1°C (“Low” treatment) and 37.8°C (“High”), and compared those to naturally incubation (“Natural”) eggs.” Once hatched, we cross‐fostered experimentally incubated nestlings to non‐natal nests with nestlings of similar age in an effort to disentangle genetic and parental effects from our experimental manipulation. We hypothesized that incubation temperature manipulations would differentially impact development and probability of survival in nestling robins. Specifically, we predicted that (1) incubation duration would vary inversely with incubation temperature, (2) nestlings incubated at experimentally controlled (see below for details on temperature selections), suboptimal temperatures would be smaller and exhibit reduced survival compared to naturally incubated individuals, and (3) individuals incubated at “High” temperature conditions would not differ in survival or development from those incubated naturally.

## 2 METHODS

### 2.1 Study site and species

The study was conducted on an 8 ha mixed coniferous tree farm located near Urbana, IL, USA (40°02′N, 88°10′W), during the 2015 and 2016 breeding seasons (1 March–30 July). The site consisted mainly of eastern white pine (*Pinus strobus*), white fir (*Abies concolor*), Balsam fir (*Abies balsamea*), Fraser fir (*Abies fraseri*), Douglas fir (*Pseudotsuga menziesii*), and Scots pine (*Pinus sylvestris*) ranging in age from newly planted saplings to 16 years old. Trees were planted in discrete rows which allowed for rapid and thorough nest searching. Robins are an appropriate study species for this work because they are found in high densities, are multibrooded, exhibit uniparental incubation, and are resilient to research manipulation. We located nests by systematically searching all trees on the site every other day. Nests found during the building and laying periods were checked every day to ensure an accurate clutch completion date. All other nest‐checks were conducted every other day to minimize disturbance to the birds, while still obtaining high‐resolution information on fate. Incubating birds typically returned to the nest within 2–3 min following a nest‐check.

### 2.2 Incubation patterns and analysis

To determine the temperature and incubation patterns of robin nests, we placed a small metallic temperature logger among the eggs (Thermochron iButtons DS1921G, Maxim, San Jose, CA, USA) on the day of clutch completion remaining in the nest until hatch. We affixed iButtons to the nests using a combination of Velcro^®^, a shirt button, and pliable wiring so as to be comparable to the eggs in both height and position within the nest and ensure direct contact with the brood patch. This setup ensured that iButton placement within the nest was consistent throughout incubation, and prevented females from removing or burying them in the nest. iButtons were also colored blue using a permanent marker that closely resembled the color of robin eggs. Temperature was recorded at 2‐min intervals, and the data were downloaded and iButtons reprogrammed about every 2–2.5 days. iButtons have some limitations because they typically cool and warm faster than eggs (Smith, Cooper, & Reynolds, 2015) and may thus provide slight temperature biases depending on the female robin's on‐nest‐to‐off‐nest bout ratio. We are using the iButton temperature data here for descriptive purposes; we were interested in examining the range of variation in temperatures experienced in natural nests rather than making precise inferences about the temperatures experienced by eggs in those nests. We used Rhythm 1.1 (Cooper & Mills, 2005) to transform raw iButton data into usable sound files and to automate selections of on‐ and off‐bouts during the incubation period. Raven Pro 1.4 (Bioacoustics Research Program, Cornell University, Ithaca, NY, USA) was used to visually assess accuracy of automated selection generated by Rhythm and to classify selections (i.e., on‐ vs. off‐bouts). These processing steps allowed us to estimate both temperature and duration of on‐ and off‐bouts for naturally incubated eggs.

### 2.3 Egg collection, incubation, and cross‐fostering

Nests that were located during the building or laying phase and that contained ≥3 eggs were deemed suitable for egg collection and were considered focal nests. Prior to the onset of incubation, we randomly selected, removed, and marked one egg from each focal nest. Eggs were placed in cotton‐lined 50‐ml Corning^™^ Falcon^™^ tubes for transport to the laboratory. Collected eggs were randomly placed into one of two temperature categories: 36.1°C (presumed suboptimal incubation temperature; Lundy, 1969; designated as “Low”) and 37.8°C (presumed optimal incubation temperature; Kuehler & Good, 1990; designated as “High”) both set to 60%–65% relative humidity (Kuehler & Good, 1990). Early on in the experiment, our Low incubation temperature was 35°C, but during the initial round of egg collections, it became evident that 35°C was too low for robin eggs (0% hatching success, *n *=* *11). Using both an ovascope (Brinsea OvaScope Egg Viewer, Titusville, FL, USA) and by dissecting eggs, we determined that hatching failure was caused by embryonic death late in development, possibly caused by nutritional stress or delayed development of the hatching muscle (Olson, Vleck, & Adams, 2008; Olson, Vleck, & Vleck, 2006). At this point, we increased the Low temperature to 36.1°C. Incubators (Turn X7, Lyons USA, Chula Vista, CA, USA) were housed at the University of Illinois, where they were closely monitored to ensure proper incubation temperature and humidity throughout the experiment. We monitored progression of embryonic development during incubation using an ovascope. Initially, we placed eggs in an incubator with egg‐turning capabilities mimicking natural turning behavior during the incubation period. Two days prior to hatching, we transferred the eggs to a nonturning incubator to ensure a safe hatching environment. Once a nestling hatched, we recorded its mass (only in 2016), marked it using a nontoxic permanent marker, transferred it to the field in a small cooler warmed with rechargeable hand‐warmers, and placed it in a non‐natal nest with two or three same‐age nestlings.

### 2.4 Nestling sampling

Experimental nestlings were re‐marked every other day with a nontoxic permanent marker until banding to ensure accurate identification throughout the experiment. On day 7 posthatch, we banded each experimentally incubated nestling (hereafter “focal nestling”) and a randomly selected naturally incubated nestling from the foster nest (hereafter “foster nest‐mate”). On days 7 and 10 posthatch, we recorded the mass and measured wing and tarsus length for each focal nestling and its associated foster nest‐mate. We also collected a small blood sample (<5% blood volume) from both chicks prior to and following the administration of a standardized 30‐min stress protocol (Breuner, Wingfield, & Romero, 1999) on days 7 and 10 posthatch as part of another study, after which nestlings were returned to the nest. Due to time constraints, only the focal nestling and foster nest‐mate were sampled and banded.

### 2.5 Statistical methods

We estimated baseline hatching success for robin eggs in our study population using only those nests that were not manipulated (i.e., no egg removed or added, and no iButton). We used generalized linear mixed models (SAS PROC GLIMMIX; binomial distribution, logit link function) to estimate the probability of a nestling surviving to day 7 or 10 posthatch for a given treatment including nest identity as a random effect. We examined morphological differences among temperature categories (Low, High, Natural) using a general linear mixed model (SAS PROC MIXED), as well as differences between a given focal nestling and its foster nest‐mate using a paired t‐test (SAS PROC TTEST) to control for parental effects (e.g., provisioning and nestling brooding). We used change in size between days 7 and 10 posthatch as a rough estimate of growth for each morphological trait and compared growth estimates among temperature categories (Low, High, Natural) using a general linear mixed model. Clutch survival (total and partial clutch loss) was estimated using generalized linear mixed models (binomial distribution, logit link function) including a random effect of nest or nestling identity in all models when appropriate. All statistical tests were performed using SAS 9.4.

## 3 RESULTS

### 3.1 Hatching success and incubation

**Figure 2:**
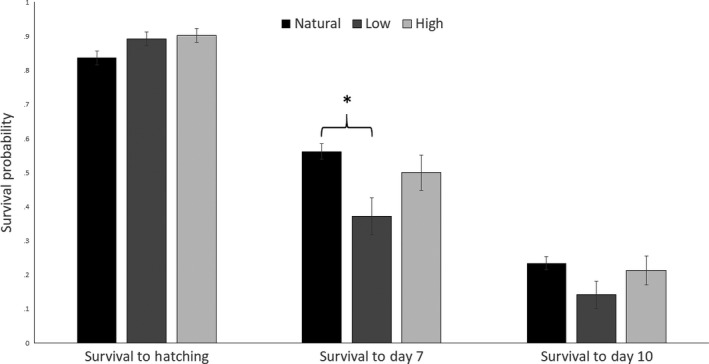
Probability of American robin (*Turdus migratorius*) egg survival to hatching, posthatch day 7, and posthatch day 10 in two experimental incubation treatments (±*SE*; Low, 36.1°C; High 37.8°C) as well as nonmanipulated nests (Natural) during the breeding seasons of 2015 and 2016. Hatching: Low: *n *=* *65; High: *n *=* *61; Natural: *n *=* *265; Day 7: Low: *n *=* *14; High: *n *=* *23; Natural: *n *=* *148; Day 10: Low: *n *=* *8; High: *n *=* *18; Natural: *n *=* *105. Asterisk denotes statistical significance (*p *≤* *.05)

**Figure 3:**
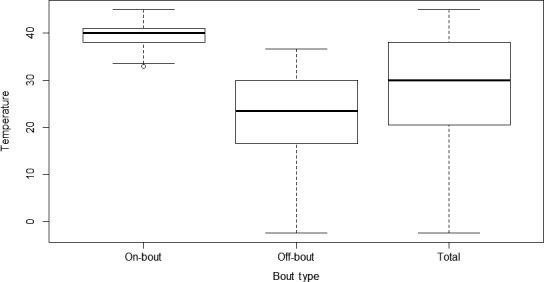
Incubation temperatures for nonmanipulated American robin (*Turdus migratorius*) nests (*n *=* *51) in 2015 and 2016. The horizontal line within the box indicates the median value, boundaries of the box indicate the 25th and 75th percentiles, respectively, and the whiskers indicate the highest and lowest values in the dataset

Across both sampling years, we located and monitored a total of 339 nests. Hatching success for nonmanipulated nests (*n *=* *87) was 85.2% (226 eggs hatched of a total of 265 eggs laid, excluding clutches/eggs lost due to predation or researcher manipulation). Hatching success for eggs from the artificial incubators was slightly higher (Low treatment [36.1°C; *n *=* *65]: 89.2%; High treatment [37.8°C; *n *=* *61]: 90.2%), but there were no significant differences among temperature categories (*p *=* *.452, Figure 2). Additionally, as mentioned above, hatching success for the initial Low treatment of 35.0°C was 0% (*n *=* *11). Following the onset of incubation, temperatures in nonmanipulated nests varied widely from a low of −2.4°C to a high of 45.0°C during extreme periods, but tended toward temperatures within a range of more optimal development (37–38°C; Table 1; Figure 3).

**Table 1:** Descriptive statistics for incubation patterns of American robins (*Turdus migratorius*) breeding in central Illinois, USA (*n *=* *51). Data were collected using metallic temperature probes (iButtons). Each iButton was preprogrammed to collect data every 2 min, removed from the nest every 2 days, and exchanged with a newly programmed iButton

Parameter	Mean (*SE*)
On‐bout duration (min)	33.7 (0.95)
Off‐bout duration (min)	20.6 (0.40)
On‐bout temperature (°C)	39.3 (0.24)
Off‐bout temperature (°C)	22.9 (1.03)
Overall mean temperature (°C)	28.3 (0.72)

**Figure 4:**
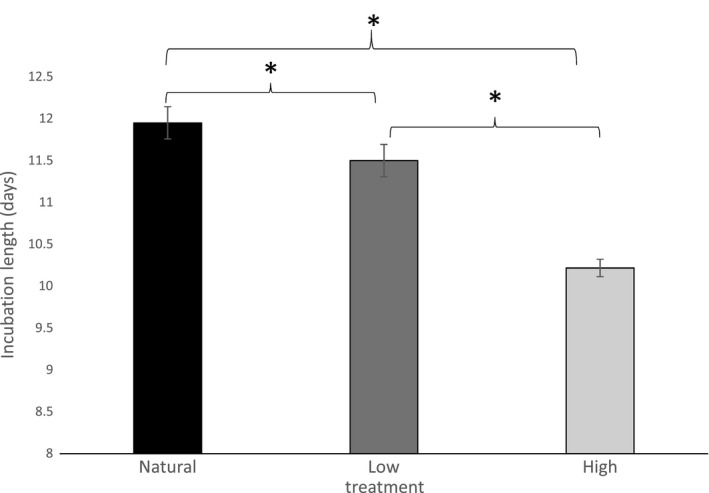
Average length of incubation period (days; ±*SE*) for naturally incubated American robin (*Turdus migratorius*) eggs, and two experimental treatments: Low (36.1°C) and High (37.8°C) during the 2015 and 2016 breeding seasons. Natural: *n *=* *265; Low: *n *=* *65; High: *n *=* *61. Asterisk denotes statistical significance (*p *≤* *.05)

Artificial incubation significantly decreased the length of the incubation period compared to natural nests (*F*_2,124_ = 31.5; *p *<* *.0001; Figure 4). Length of the incubation period was more similar between Low treatment (11.50 days) and naturally incubated (11.95 days) eggs (differing by 0.45 days, *p *=* *.038) than between those incubated in the High treatment (10.22 days) and naturally incubated eggs (differing by 1.73 days, *p *<* *.0001), although both treatments differed from the naturally incubated nestlings. High‐treatment eggs hatched significantly more quickly compared with Low‐treatment eggs (differing by 1.28 days; *p *=* *.038).

### 3.2 Nestling survival and morphology

Nestling mass at hatch did not differ between experimental incubation treatment (*F*_1,47_ = 0.72; *p *=* *.40). Incubation category (Low, High, Natural) had a significant effect on survival to day 7 posthatch, but not on survival to day 10 posthatch (day 7: *F*_2,623_ = 4.83; *p *=* *.008; day 10: *F*_2,623_ = 1.64; *p *=* *.195). Naturally incubated nestlings had a higher probability of survival to days 7 and 10 posthatch compared with both High‐ and Low‐treatment nestlings (Figure 2). High‐treatment nestlings exhibited intermediate levels of survival to days 7 and 10 posthatch, with marginally significantly higher survival compared with Low‐treatment nestlings, and nonsignificantly lower survival than naturally incubated nestlings (day 7: High vs. Low: *p *=* *.093; High vs. Natural: *p *=* *.275; day 10: High vs. Low: *p *=* *.226; High vs. Natural: *p *=* *.664; Figure 2). Nests that received an artificially incubated nestling were significantly more likely to experience total clutch loss than nonmanipulated nests (*F*_1,457_ = 3.84; *p *=* *.050). Rates of total clutch loss did not differ between the two experimental treatments (36.1°C and 37.8°C; *F*_1,456_ = 0.43; *p *=* *.664). There was, however, an effect of treatment on partial clutch loss (defined as any nest that lost 1 or more nestling(s) while still fledging at least 1 young), in which nests that contained a Low‐treatment nestling were more likely to experience partial clutch loss than nests with a High‐treatment nestling (75% of nests in Low treatment experienced partial clutch loss compared with 15% loss from High treatment and 12% loss from unmanipulated Natural nests; *F*_1,12_ = 4.56; *p *=* *.052).

**Figure 5:**
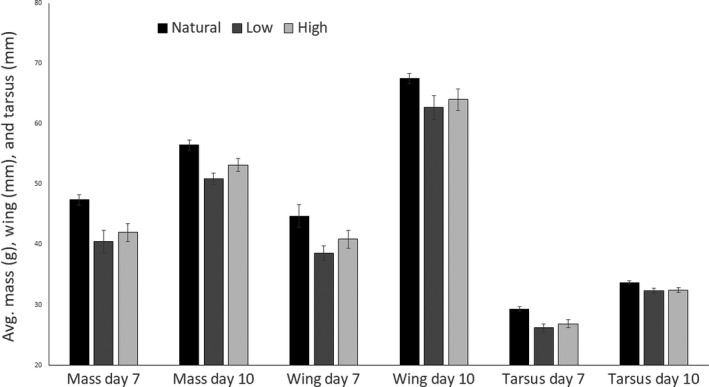
Mean mass (g), relaxed wing chord (mm), and tarsus length (mm ± *SE*) of experimentally (Low, 36.1°C; High 37.8°C) and naturally incubated American robin (*Turdus migratorius*) nestlings on days 7 and 10 during the nestling period of the 2015 and 2016 breeding seasons. Day 7: Low: *n *=* *14; High: *n *=* *23; Natural: *n *=* *37; day 10: Low: *n *=* *8; High: *n *=* *18; Natural: *n *=* *26. There was no significant effect of incubation treatment on mean mass, wing chord, or tarsus length at day 7 or day 10

**Figure 6:**
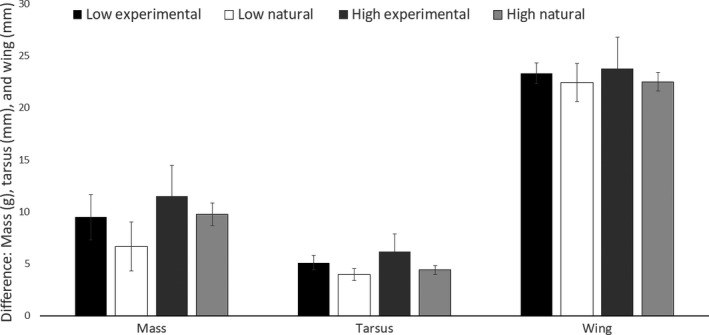
Comparison of change in size (days 10–7) between each focal nestling (Low, 36.1°C, black bars; High, 37.8°C, dark gray bars) and their paired naturally incubated foster nest mate (paired with Low = white, paired with High = light gray, ±*SE*) in American robin (*Turdus migratorius*) nestlings during the 2015 and 2016 breeding seasons. Low: *n *=* *8; High: *n *=* *18

On days 7 and 10 posthatch, mean mass, wing, and tarsus length were smallest for Low‐treatment nestlings, and largest for Natural nestlings (Figure 5). Focal chicks from both experimental treatments were significantly smaller than their foster nest mates in mass and tarsus length on day 7 posthatch, but that difference only persisted for the focal chicks from the High treatment to day 10 posthatch (Table 2). On day 10 posthatch, focal nestlings from the Low treatment no longer differed significantly in mass or tarsus length from their foster nest‐mate (Table 2). Focal nestlings from the Low treatment had significantly shorter wings than their foster nest mates on day 7 and day 10 posthatch (Table 2). High‐treatment nestlings and their foster nest‐mate did not differ in wing length at either time point (Table 2). Nestlings that hatched from artificially incubated eggs (either experimental treatment) tended to exhibit greater growth than their foster nest‐mate, but growth was not significantly different for any of the morphometrics (Table 2; Figure 6).

**Table 2:** Results of pairwise comparisons between each focal nestling (i.e., chicks from the Low [36.1°C] and High [37.8°C] temperature treatments) and its foster nest‐mate (a naturally incubated chick) on days 7 and 10 posthatch, and the difference between each measurement from days 7 to 10 posthatch (growth). Significant pairwise differences (*p *≤* *.05) bolded

	Day 7			Day 10			Difference		
	*t*	*df*	*p*	*t*	*df*	*p*	*t*	*df*	*p*
Low—Tarsus	**−2.99**	**13**	**.010**	−1.18	7	.278	−1.28	7	.243
Low—Mass	**−3.00**	**13**	**.010**	−1.2	7	.270	−0.80	7	.452
Low—Wing	**−5.21**	**13**	**<.001**	**−2.84**	**7**	**.025**	−0.70	7	.504
High—Tarsus	**−3.33**	**20**	**.003**	−**2.62**	**14**	**.020**	−0.27	13	.790
High—Mass	**−3.09**	**20**	**.006**	**−3.06**	**14**	**.009**	−1.41	13	.183
High—Wing	−1.24	20	.229	−1.2	14	.250	−0.44	13	.668

## 4 DISCUSSION

Our results demonstrate that differences in incubation temperature within the natural range of variation can have large‐scale effects on growth and survival in robin nestlings. These effects manifested as reduced incubation duration for both experimental temperature categories compared with naturally incubated eggs, as well as lower survival, shorter mean wing length, and smaller mean mass and tarsus length at Low temperatures (i.e., 36.1°C) compared to nestlings from High (i.e., 37.8°C) and naturally incubated nestlings. These results complement prior work documenting adverse effects of low incubation temperatures in cavity‐nesting wild birds (DuRant et al., 2010; Nord & Nilsson, 2011; Reid et al., 2002), but also provide novel insight into the importance of incubation temperature for open‐cup nesting species.

Similar to previous research (DuRant et al., 2010; Nord & Nilsson, 2011), we found that the incubation period was shortest for eggs artificially incubated at higher experimental temperatures. While more rapid rates of development may be beneficial (e.g., when risk of nest predation is high), there is likely some optimal rate of development above which the offspring suffer deleterious effects. Nestlings from both experimental treatments were generally smaller for all three measured traits compared with nestlings from naturally incubated nests. Our results corroborate findings from previous research on blue tit nestlings (*Cyanistes caeruleus*) in which nestlings from the lower temperature treatment tended to be smaller than those from the higher temperature treatment, although not significantly so (Nord & Nilsson, 2011). We predicted that Low‐treatment nestlings would be smaller than High‐treatment nestlings, and High‐treatment nestlings would not differ in size (mass, wing and tarsus length) from naturally incubated nestlings given that ~38°C is presumed to be optimal based on experimental work with a congener of the robin (Kuehler & Good, 1990). When comparing raw means across treatments, we did not find significant differences in mass, wing, or tarsus length, but when we restricted comparisons to focal chicks and their foster nest‐mates (i.e. within nests), we found that the experimental chicks from either temperature treatment were significantly smaller than their naturally incubated foster nest mates on day 7 posthatch, and that nestlings from the High treatment remained significantly smaller on day 10 posthatch. Controlling for variation among nests allowed us to isolate the effects of the incubation treatment.

The smaller size of experimentally incubated nestlings may have been caused by several factors. Experimentally incubated eggs were exposed to relatively constant temperatures, whereas naturally incubated eggs experienced temperature fluctuations throughout the incubation period. These fluctuations may play an important role in proper embryonic development and can potentially impact nestling traits important for future development, survival, and reproduction (DuRant, Hopkins, Hepp, & Walters, 2013). Indeed, previous research suggests that slower development time is linked to intrinsic mechanisms (e.g., rate of yolk consumption) and can yield long‐term benefits for offspring quality (Metcalfe & Monaghan, 2003). Therefore, by maintaining eggs in a state of high and constant incubation temperature (particularly in the High treatment and to a lesser extent in the Low treatment), we potentially reduced offspring quality by forcing more rapid rates of embryonic development (Vleck & Vleck, 1996). This more rapid development is likely linked to elevated metabolic activity, which requires greater energy input (Vleck & Vleck, 1996). Females deposit finite energy stores within the egg, and after the eggs are laid, no further organic nutrients can be made available to the developing embryo until hatching. The amount of resources deposited into the egg represents a trade‐off in investment between current and future reproduction, but on average should provide sufficient resources for the embryo under normal incubation conditions. We found that naturally incubated eggs experienced temperatures well above our High treatment, but these were for brief periods of time, and were offset by off‐bout periods (Figure 3). We also found that robin embryos were capable of developing under low‐temperature conditions but never successfully hatched at our initial Low‐temperature treatment (35°C), indicating that there is a fine balance between the rate of development of robin embryos and the resources deposited within the eggs. It is worth noting that domestic chicken eggs are typically incubated at high (37.8°C ± 0.3) and constant incubation temperatures for optimal hatching success and chick quality (Barott, 1937), although some growers have utilized incubators with cyclical reductions in temperatures, thereby mimicking natural fluctuations (reviewed in: Kosin, 1964). To what extent optimal incubation patterns differ between precocial and altricial species, or between Galliformes and Passeriformes are unclear, but there is substantial variation among species in parental incubation behavior (Martin, Oteyza, Boyce, Lloyd, & Ton, 2015). One general trend is that species with precocial young tend to exhibit higher attentiveness during incubation and have higher constancy (i.e., the amount of the time the eggs are in contact with an adult relative to the entire incubation period) compared to species with altricial young (Deeming, 2002a, 2002b).

We found a significant reduction in nestling survival to day 7 between experimental and natural chicks, and the primary cause of this reduction was depredation of all nest contents (i.e., total clutch loss). This differential survival suggests that the experimental nestlings were in some way influencing the fate of the entire nest, possibly through increased begging behavior and/or increased adult provisioning (Martin, Scott, & Menge, 2000). For example, research on seabird chicks suggests that during periods of prenatal stress, such as decreased incubation temperatures, there is an increase in postnatal stress hormone (corticosterone) concentration. Increased corticosterone concentrations are linked to more aggressive begging and elevated begging rates (Kitaysky, Kitaiskaia, Piatt, & Wingfield, 2003), and changes in the begging behavior of one chick can impact begging behavior of all the chicks in the nest (Elderbrock, Small, & Schoech, 2017). If experimental nestlings are begging more and/or if they induce their nest mates to beg more, nest‐predation rates can increase as a result of predators cuing in on begging calls (Haskell, 1994; Leech & Leonard, 1997), or via increased provisioning rates of the adults (Martin et al., 2000). In this manner, differences in incubation temperatures experienced by the experimental chick can translate into differences in total nest survival rates. More research is needed to explore the relationship between incubation conditions and nestling behavior and the potential downstream impacts on predation risk.

We also found a nonsignificant difference in nestling survival between experimental treatments, which was driven by significantly higher rates of selective loss of the experimental chick for Low‐treatment nestlings compared with High‐treatment nestlings. This high rate of selective loss for the Low‐treatment nestlings compared with High‐treatment and Natural nestlings suggests that the selective loss of the Low‐treatment focal nests is not a product of artificial incubation, but was related to the differences in the incubation temperature. Within the framework of “parental optimism” (sensu Mock & Forbes, 1995), parents will selectively favor the larger and therefore more robust offspring within the clutch, hedging against resource uncertainty. Therefore, the disappearance of the smaller Low‐treatment chick likely reflects death due to poor condition rather than selective partial nest‐predation (Forbes & Glassey, 2000; Mock & Forbes, 1995) and indicates that incubation temperature can have detrimental developmental effects that may be too great for nestlings to overcome. In addition, we found no evidence that experimental nestlings remained in the nest longer than natural nestlings (E. A. Ospina, personal observation), indicating that they were unable to compensate for their poor start by extending the nestling period (Metcalfe & Monaghan, 2001).

Another possible strategy for the smaller experimental nestlings would be to invest in compensatory growth, although there is a suite of negative effects related to this strategy (Metcalfe & Monaghan, 2001). However, based on our longitudinal morphometric data, we believe it is unlikely that experimental nestlings exhibited high enough rates of compensatory growth between day 10 and 13 to catch up to the Natural chicks (Table 2; Figure 4). Robins typically fledge at 13 days posthatch, and experimental chicks likely would have fledged at a smaller size than Natural chicks. Smaller individuals often experience lower postfledging survival (Magrath, 1991; Vitz & Rodewald, 2011), so incubation temperature may continue to impact mortality after fledging. Fledglings that leave the nest in better condition may have superior foraging skills, better ability to evade predators, and greater capacity to cope with adverse environmental conditions (Jones, Ward, Benson, & Brawn, 2017; Vitz & Rodewald, 2011). Additionally, previous research in marsh tits (*Parus palustris*) suggests that smaller nestlings within a brood will prioritize alternate growth strategies (wing development) when faced with sibling competition from dominant (i.e., older) nest mates (Nilsson & Svensson, 1996). The smaller individuals from the Low treatment in our study may have been constrained by their exposure to suboptimal incubation temperatures, forcing them to invest in somatic growth to stay competitive with their nest mates, potentially at the expense of their postfledging survival. In contrast, nestlings from the High treatment, which tended to be larger than the Low‐treatment nestlings, did not experience this same reduction in wing development as compared to their naturally incubated foster nest mate. It is possible that the High‐treatment nestlings may have allocated available resources to wing development to increase their postfledging survival probability.

The large effects on survival and development resulting from relatively small differences in incubation conditions underscore the potential for landscape‐level changes to have population‐level impacts on wild birds. For example, habitat fragmentation, increased exposure to predators, and urbanization may impact incubation behavior (Baudains & Lloyd, 2007; Crooks & Soulé, 1999; Weston & Elgar, 2007), and our results suggest that such changes could fundamentally impact the phenotypes and ultimately the fitness of these birds. Indeed, previous research suggests that incubating females are capable of altering their incubation behavior in response to perceived predation risk (Fontaine & Martin, 2006; Ghalambor & Martin, 2002; LaManna & Martin, 2016), and given the potential ramifications of changes in incubation conditions, this is a field that warrants further investigation.

Incubation temperature plays a large role in the pre‐ and posthatch development of young birds and can have important consequences for chick survival. Nestlings from experimentally manipulated incubation treatments (High and Low) exhibited a contracted incubation period, smaller body size, lower nestling survival, and presumably smaller fledgling size leaving the nest as compared to naturally incubated nestlings. Although this work provides needed baseline information on the effects of changing incubation temperature for altricial, open‐cup nesting species, future research should examine additional factors, including temperature fluctuations and modifications to natural nests to influence incubation temperature. This research provides compelling evidence linking early developmental experiences to nestling development, condition, and survival.

## CONFLICT OF INTEREST

None declared.

## AUTHOR CONTRIBUTIONS

EAO, LM, and TJB conceived and designed this study. EAO and LM executed the study. EAO performed statistical analyses with guidance from LM and TJB who also provided extensive editorial comments.

## References

[ece33911-bib-0001] Andrews, R. M. , Mathies, T. , & Warner, D. A. (2000). Effect of incubation temperature on morphology, growth and survival of juvenile *Sceloporus undulatus* . Herpetological Monographs, 14, 420–431. https://doi.org/10.2307/1467055

[ece33911-bib-0002] Ardia, D. R. , Pérez, J. H. , & Clotfelter, E. D. (2010). Experimental cooling during incubation leads to reduced innate immunity and body condition in nestling tree swallows. Proceedings of the Royal Society of London B: Biological Sciences, 277, 1881–1888. https://doi.org/10.1098/rspb.2009.2138 10.1098/rspb.2009.2138 PMC2871872 20147326

[ece33911-bib-0003] Barott, H. G. (1937). Effects of temperature, humidity and other factors on hatch of eggs and on energy metabolism of chick embryos. USDA Technical Bulletin No. 553.

[ece33911-bib-0004] Baudains, T. P. , & Lloyd, P. (2007). Habituation and habitat changes can moderate the impacts of human disturbance on shorebird breeding performance. Animal Conservation, 10, 400–407. https://doi.org/10.1111/j.1469-1795.2007.00126.x

[ece33911-bib-0005] Breuner, C. W. , Wingfield, J. C. , & Romero, L. M. (1999). Diel rhythms of basal and stress‐induced corticosterone in a wild, seasonal vertebrate, Gambel's white‐crowned sparrow. Journal of Experimental Zoology, 284, 334–342. https://doi.org/10.1002/(ISSN)1097-010X 10404125

[ece33911-bib-0006] Bull, J. J. (1980). Sex determination in reptiles. Quarterly Review of Biology, 55, 3–21. https://doi.org/10.1086/411613

[ece33911-bib-0007] Burger, J. (1989). Incubation temperature has long‐term effects on behaviour of young pine snakes (*Pituophis melanoleucus*). Behavioral Ecology and Sociobiology, 24, 201–207. https://doi.org/10.1007/BF00295199

[ece33911-bib-0008] Burger, J. (1990). Effects of incubation temperature on behavior of young black racers (*Coluber constrictor*) and kingsnakes (*Lampropeltis getulus*). Journal of Herpetology, 24, 158–163. https://doi.org/10.2307/1564223

[ece33911-bib-0009] Carey, C. (1980). The ecology of avian incubation. BioScience, 30, 819–824. https://doi.org/10.2307/1308374

[ece33911-bib-0010] Conway, C. J. , & Martin, T. E. (2000). Evolution of passerine incubation behavior: Influence of food, temperature, and nest predation. Evolution, 54, 670–685. https://doi.org/10.1111/j.0014-3820.2000.tb00068.x 10937242 10.1111/j.0014-3820.2000.tb00068.x

[ece33911-bib-0011] Cooper, C. B. , & Mills, H. (2005). New software for quantifying incubation behavior from time‐series recordings. Journal of Field Ornithology, 76, 352–356. https://doi.org/10.1648/0273-8570-76.4.352

[ece33911-bib-0012] Crooks, K. R. , & Soulé, M. E. (1999). Mesopredator release and avifaunal extinctions in a fragmented system. Nature, 400, 563–566. https://doi.org/10.1038/23028

[ece33911-bib-0013] Deeming, D. C. (2002a). Importance and evolution of incubation in avian reproduction In Deeming D. C. (Ed.), Avian incubation: Behaviour, environment, and evolution (pp. 1–7). Oxford, UK: Oxford University Press.

[ece33911-bib-0014] Deeming, D. C. (2002b). Behaviour patterns during incubation In Deeming D. C. (Ed.), Avian incubation: Behaviour, environment, and evolution (pp. 63–87). Oxford, UK: Oxford University Press.

[ece33911-bib-0015] DuRant, S. E. , Hepp, G. R. , Moore, I. T. , Hopkins, B. C. , & Hopkins, W. A. (2010). Slight differences in incubation temperature affect early growth and stress endocrinology of wood duck (*Aix sponsa*) ducklings. Journal of Experimental Biology, 213, 45–51. https://doi.org/10.1242/jeb.034488 20008361 10.1242/jeb.034488

[ece33911-bib-0016] DuRant, S. E. , Hopkins, W. A. , Hepp, G. R. , & Walters, J. R. (2013). Ecological, evolutionary, and conservation implications of incubation temperature‐dependent phenotypes in birds. Biological Reviews, 88, 499–509. https://doi.org/10.1111/brv.12015 23368773 10.1111/brv.12015

[ece33911-bib-0017] Elderbrock, E. K. , Small, T. W. , & Schoech, S. J. (2017). Influence of corticosterone treatment on nestling begging in Florida scrub‐jays (*Aphelocoma coerulescens*). General and Comparative Endocrinology, pii: S0016‐6480(16)30365‐3. https://doi.org/10.1016/j.ygcen.2017.12.003 10.1016/j.ygcen.2017.12.003 29217466

[ece33911-bib-0018] Fontaine, J. J. , & Martin, T. E. (2006). Parent birds assess nest predation risk and adjust their reproductive strategies. Ecology Letters, 9, 428–434. https://doi.org/10.1111/j.1461-0248.2006.00892.x 16623728 10.1111/j.1461-0248.2006.00892.x

[ece33911-bib-0019] Forbes, S. , & Glassey, B. (2000). Asymmetric sibling rivalry and nestling growth in red‐winged blackbirds (*Agelaius phoeniceus*). Behavioral Ecology and Sociobiology, 48, 413–417. https://doi.org/10.1007/s002650000239

[ece33911-bib-0020] Ghalambor, C. K. , & Martin, T. E. (2002). Comparative manipulation of predation risk in incubating birds reveals plasticity of responses. Behavioral Ecology, 13, 101–108. https://doi.org/10.1093/beheco/13.1.101

[ece33911-bib-0021] Haskell, D. (1994). Experimental evidence that nestling begging behavior incurs a cost due to nest predation. Proceedings of the Royal Society of London B: Biological Sciences, 257, 161–164. https://doi.org/10.1098/rspb.1994.0110

[ece33911-bib-0022] Heenan, C. B. (2013). An overview of the factors influencing the morphology and thermal properties of avian nests. Avian Biology Research, 6, 104–118. https://doi.org/10.3184/003685013X13614670646299

[ece33911-bib-0023] Hepp, G. , Kennamer, R. A. , & Johnson, M. H. (2006). Maternal effects in wood ducks: Incubation temperature influences incubation period and neonate phenotype. Functional Ecology, 20, 307–314.

[ece33911-bib-0024] Hulet, R. , Gladys, G. , Hill, D. , Meijerhod, R. , & El‐Shiekh, T. (2007). Influence of egg shell embryonic incubation temperature and broiler breeder flock age on posthatch growth performance and carcass characteristics. Poultry Science, 86, 408–412. https://doi.org/10.1093/ps/86.2.408 10.1093/ps/86.2.408 17234858

[ece33911-bib-0025] Jones, T. M. , Ward, M. P. , Benson, T. J. , & Brawn, J. D. (2017). Variation in nestling body condition and wing development predict cause‐specific mortality in fledgling dickcissels. Journal of Avian Biology, 48, 439–447. https://doi.org/10.1111/jav.01143

[ece33911-bib-0026] Kitaysky, A. S. , Kitaiskaia, E. V. , Piatt, J. F. , & Wingfield, J. C. (2003). Benefits and costs of increased levels of corticosterone in seabird chicks. Hormones and Behavior, 43, 140–149. https://doi.org/10.1016/S0018-506X(02)00030-2 12614644 10.1016/s0018-506x(02)00030-2

[ece33911-bib-0027] Kosin, I. L. (1964). Recent research trends in hatchability—Related problems of the domestic fowl. World's Poultry Science Journal, 20, 254–268. https://doi.org/10.1079/WPS19640033

[ece33911-bib-0028] Kuehler, C. , & Good, J. (1990). Artificial incubation of bird eggs at the Zoological Society of San Diego. International Zoo Yearbook, 29, 118–136.

[ece33911-bib-0029] Lack, D. (1954). The natural regulation of animal numbers. Oxford, UK: Oxford University Press.

[ece33911-bib-0030] LaManna, J. A. , & Martin, T. E. (2016). Costs of fear: Behavioural and life‐history responses to risk and their demographic consequences vary across species. Ecology Letters, 19, 403–413. https://doi.org/10.1111/ele.12573 26900087 10.1111/ele.12573

[ece33911-bib-0031] Leech, S. M. , & Leonard, M. L. (1997). Begging and the risk of predation in nestling birds. Behavioral Ecology, 8, 644–646. https://doi.org/10.1093/beheco/8.6.644

[ece33911-bib-0032] Leksrisompong, N. , Romero‐Sanchez, H. , Plumstead, P. W. , Bannan, K. E. , & Brake, J. (2007). Broiler incubation. 1. Effect of elevated temperature during late incubation on body weight and organs of chicks. Poultry Science, 86, 2685–2691. https://doi.org/10.3382/ps.2007-00170 10.3382/ps.2007-00170 18029817

[ece33911-bib-0033] Lundy, H. (1969). A review of the effects of temperature, humidity, turning and gaseous environment in the incubator on the hatchability of the hen's egg In Freeman B. M. (Ed.), The fertility and hatchability of the hen's egg (pp. 143–176). Edinburg, UK: Oliver and Boyd.

[ece33911-bib-0034] Magrath, R. D. (1991). Nestling weight and juvenile survival in the blackbird, *Turdus merula* . Journal of Animal Ecology, 60, 335–351. https://doi.org/10.2307/5464

[ece33911-bib-0035] Martin, T. E. (2002). A new view of avian life‐history evolution tested on an incubation paradox. Proceedings of the Royal Society B: Biological Sciences, 269, 309–316. https://doi.org/10.1098/rspb.2001.1879 11839200 10.1098/rspb.2001.1879 PMC1690888

[ece33911-bib-0036] Martin, T. E. , Boyce, A. J. , Fierro‐Calderón, K. , Mitchell, A. E. , Armstad, C. E. , Mouton, J. C. , … Evertius, E. (2017). Enclosed nests may provide greater thermal than nest predation benefits compared with open nests across latitudes. Functional Ecology, 31, 1231–1240. https://doi.org/10.1111/1365-2435.12819

[ece33911-bib-0037] Martin, T. E. , Oteyza, J. C. , Boyce, A. J. , Lloyd, P. , & Ton, R. (2015). Adult mortality probability and nest predation rates explain parental effort in warming eggs with consequences for embryonic development time. American Naturalist, 186, 223–236. https://doi.org/10.1086/681986 10.1086/681986 26655151

[ece33911-bib-0038] Martin, T. E. , Scott, J. , & Menge, C. (2000). Nest predation increases with parental activity: Separating nest site and parental activity effects. Proceedings of the Royal Society of London B: Biological Sciences, 267, 2287–2293. https://doi.org/10.1098/rspb.2000.1281 10.1098/rspb.2000.1281 PMC1690815 11413645

[ece33911-bib-0039] Metcalfe, N. B. , & Monaghan, P. (2001). Compensation for a bad start: Grow now, pay later? Trends in Ecology and Evolution, 16, 254–260. https://doi.org/10.1016/S0169-5347(01)02124-3 11301155 10.1016/s0169-5347(01)02124-3

[ece33911-bib-0040] Metcalfe, N. B. , & Monaghan, P. (2003). Growth versus lifespan: Perspectives from evolutionary ecology. Experimental Gerontology, 38, 935–940. https://doi.org/10.1016/S0531-5565(03)00159-1 12954479 10.1016/s0531-5565(03)00159-1

[ece33911-bib-0041] Michels, H. , Geers, R. , & Muambi, S. (1974). The effect of incubation on temperature on pre‐ and post‐hatching development in chickens. British Poultry Science, 15, 517–523. https://doi.org/10.1080/00071667408416142 10.1080/00071667408416142 4441916

[ece33911-bib-0042] Mock, D. W. , & Forbes, L. S. (1995). The evolution of parental optimism. Trends in Ecology and Evolution, 10, 130–134. https://doi.org/10.1016/S0169-5347(00)89014-X 21236982 10.1016/s0169-5347(00)89014-x

[ece33911-bib-0043] Mousseau, T. A. , & Fox, C. W. (1998). The adaptive significance of maternal effects. Trends in Ecology and Evolution, 13, 403–407. https://doi.org/10.1016/S0169-5347(98)01472-4 21238360 10.1016/s0169-5347(98)01472-4

[ece33911-bib-0044] Nangsuay, A. , Meijerhof, R. , Van den Anker, I. , Heetkamp, M. J. W. , De Souza Morita, V. , Kemp, B. , & Van Den Brand, H. (2016). Effects of breeder age, broiler strain, and eggshell temperature on development and physiological status of embryos and hatchlings. Poultry Science, 95, 1666–1679. https://doi.org/10.3382/ps/pew080 10.3382/ps/pew080 26957632

[ece33911-bib-0045] Nice, M. M. (1957). Nesting success in altricial birds. The Auk, 74, 305–321. https://doi.org/10.2307/4081922

[ece33911-bib-0046] Nilsson, J. A. , & Svensson, M. (1996). Sibling competition affects nestling growth strategies in marsh tits. Journal of Animal Ecology, 65, 825–836. https://doi.org/10.2307/5680

[ece33911-bib-0047] Nord, A. , & Nilsson, J. A. (2011). Incubation temperature affects growth and energy metabolism in blue tit nestlings. American Naturalist, 178, 639–651. https://doi.org/10.1086/662172 10.1086/662172 22030733

[ece33911-bib-0048] Olson, C. R. , Vleck, C. M. , & Adams, D. C. (2008). Decoupling morphological development from growth in periodically cooled zebra finch embryos. Journal of Morphology, 269, 875–883. https://doi.org/10.1002/(ISSN)1097-4687 18488991 10.1002/jmor.10635

[ece33911-bib-0049] Olson, C. R. , Vleck, C. M. , & Vleck, D. (2006). Periodic cooling of bird eggs reduces embryonic growth efficiency. Physiological and Biochemical Zoology, 79, 927–936. https://doi.org/10.1086/506003 16927239 10.1086/506003

[ece33911-bib-0050] Rana, K. J. (1990). Influence of incubation temperature on *Oreochromis niloticus* eggs and fry: I. Gross embryology, temperature tolerance, and rate of embryonic development. Aquaculture, 87, 165–181. https://doi.org/10.1016/0044-8486(90)90273-P

[ece33911-bib-0051] Reid, J. M. , Monaghan, P. , & Nager, R. G. (2002). Importance and the costs of reproduction In Deeming D. C. (Ed.), Avian incubation: Behavior, environment, and evolution (pp. 314–325). Oxford, UK: Oxford University Press.

[ece33911-bib-0052] Ricklefs, R. E. (1969). An analysis of nesting mortality in birds. Smithsonian Contributions to Zoology, 9, 1–48. https://doi.org/10.5479/si.00810282.9

[ece33911-bib-0053] Shine, R. , Elphick, M. J. , & Harlow, P. S. (1997). The influence of natural incubation environments on the phenotypic traits of hatchling lizards. Ecology, 78, 2559–2568. https://doi.org/10.1890/0012-9658(1997)078[2559:TIONIE]2.0.CO;2

[ece33911-bib-0054] Skutch, A. F. (1962). The constancy of incubation. Wilson Bulletin, 74, 115–152.

[ece33911-bib-0055] Smith, J. A. , Cooper, C. B. , & Reynolds, S. J. (2015). Advances in techniques to study incubation In Deeming D. C. & Reynolds S. J. (Eds.), Nests, eggs, and incubation (pp. 179–195). Oxford, UK: Oxford University Press https://doi.org/10.1093/acprof:oso/9780198718666.001.0001

[ece33911-bib-0056] Spotila, J. R. , Zimmerman, L. C. , Binckley, C. A. , Grumbles, J. S. , Rostal, D. C. , List, Jr, A. , … Kemp, S. J. (1994). Effects of incubation conditions on sex determination, hatching success, and growth of hatchling desert tortoises, *Gopherus agassizii* . Herpetological Monographs, 8, 103–116. https://doi.org/10.2307/1467074

[ece33911-bib-0057] Stearns, S. C. (1992). The evolution of life histories. Oxford, UK: Oxford University Press.

[ece33911-bib-0058] Ton, R. , & Martin, T. E. (2017). Proximate effects of temperature versus evolved intrinsic constraints for embryonic development times among temperature and tropical songbirds. Scientific Reports, 7, 895 https://doi.org/10.1038/s41598-017-00885-3 28420877 10.1038/s41598-017-00885-3 PMC5429855

[ece33911-bib-0059] Uller, T. (2008). Developmental plasticity and the evolution of parental effects. Trends in Ecology and Evolution, 23, 432–438. https://doi.org/10.1016/j.tree.2008.04.005 18586350 10.1016/j.tree.2008.04.005

[ece33911-bib-0060] Van Damme, R. , Bauwens, D. , Braña, F. , & Verheyen, R. F. (1992). Incubation temperature differentially affects hatching time, egg survival, and hatching performance in the lizard *Podarcis muralis* . Herpetologia, 48, 220–228.

[ece33911-bib-0061] Vitz, A. C. , & Rodewald, A. D. (2011). Influence of condition and habitat use on survival of post‐fledging songbirds. The Condor, 113, 400–411. https://doi.org/10.1525/cond.2011.100023

[ece33911-bib-0062] Vleck, C. M. , & Vleck, D. (1996). Embryonic energetics In Carey C. (Ed.), Avian energetics and nutritional ecology (pp. 417–460). New York, NY: Chapman and Hall https://doi.org/10.1007/978-1-4613-0425-8

[ece33911-bib-0801] Wada, H. , Kriengwatana, B. , Allen, N. , Schmidt, K. L. , Soma, K. K. , & MacDougall‐Shackleton, S. A. (2015). Transient and permanent effects of suboptimal incubation temperatures on growth, metabolic rate, immune function and adrenocortical responses in zebra finches. Journal of Experimental Biology, 218, 2847–2855. 26206355 10.1242/jeb.114108

[ece33911-bib-0063] Weaver, I. C. G. (2009). Shaping adult phenotypes through early life environments. Birth Defects Research Part C: Embryo Today: Reviews, 87, 314–326. https://doi.org/10.1002/bdrc.20164 10.1002/bdrc.20164 19960543

[ece33911-bib-0064] Webb, D. R. (1987). Thermal tolerance of avian embryos: A review. Condor, 89, 874–898. https://doi.org/10.2307/1368537

[ece33911-bib-0065] Weston, M. A. , & Elgar, M. A. (2007). Responses of incubation hooded plovers (*Thinornis rubricollis*) to disturbance. Journal of Coastal Research, 23, 569–576. https://doi.org/10.2112/04-0151.1

