# Supplementary material for: Incubation temperature impacts nestling growth and survival in an open‐cup nesting passerine
Source: Ecol Evol. 2018 Feb 19;8(6):3270–9. doi: 10.1002/ece3.3911 (PMC5869297; doi:10.1002/ece3.3911)
Supplement: Supplementary file 1 [file ECE3-8-3270-s001.docx]

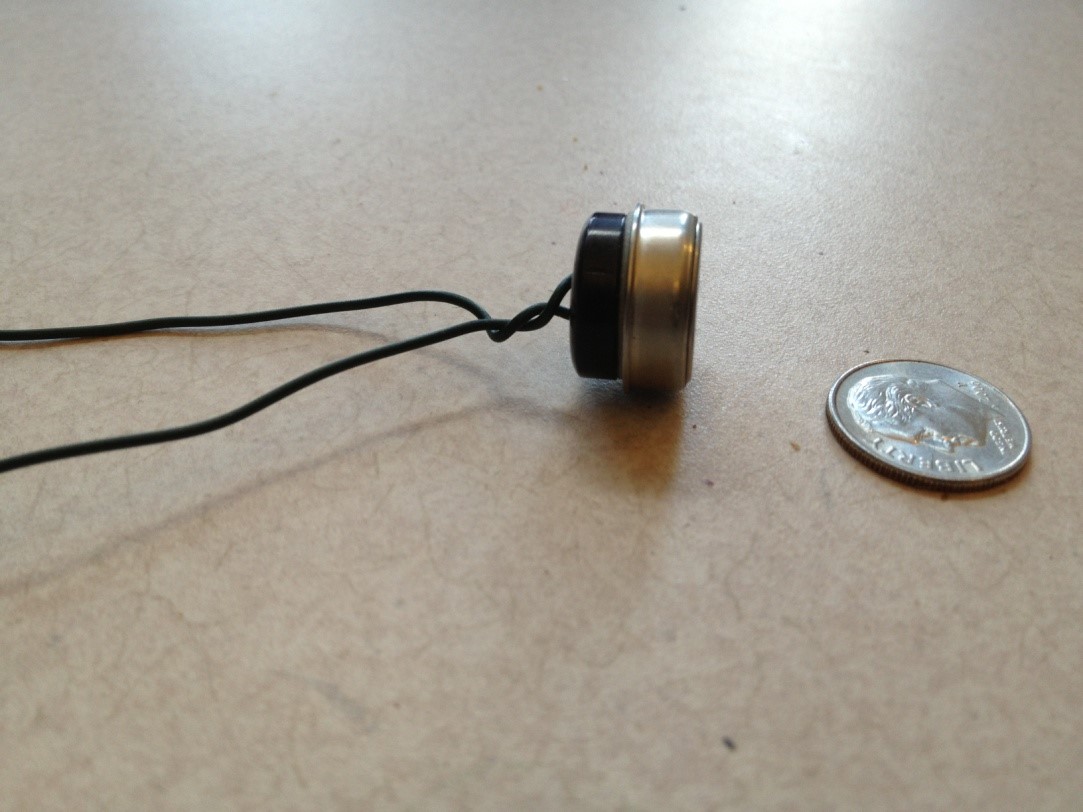


Supplemental Fig. S1. Photograph of an iButton setup with a dime for scale. Small gauge wire was threaded through a shirt button (black disk) and affixed to the iButton (silver disk) using Velcro® and super glue serving as a placeholder for the iButton within the nest through the entirety of incubation.
